# Unfolding and Aggregation of Lysozyme under the Combined Action of Dithiothreitol and Guanidine Hydrochloride: Optical Studies

**DOI:** 10.3390/ijms22052710

**Published:** 2021-03-08

**Authors:** Ruslan M. Sarimov, Vladimir N. Binhi, Tatiana A. Matveeva, Nikita V. Penkov, Sergey V. Gudkov

**Affiliations:** 1 Prokhorov General Physics Institute of the Russian Academy of Sciences, Vavilov St., 38, 119991 Moscow, Russia; rusa@kapella.gpi.ru (R.M.S.); binhi@kapella.gpi.ru (V.N.B.); pticek@yandex.ru (T.A.M.); 2 Institute of Cell Biophysics of the Russian Academy of Sciences, PSCBR RAS, Institutskaya St., 3, Pushchino, 142290 Moscow, Russia; nvpenkov@rambler.ru

**Keywords:** protein hydration shells, optical methods in biochemistry, interferometry, dynamic light scattering (DLS), UV/visible absorbance spectroscopy, analysis of protein activity

## Abstract

Using a number of optical techniques (interferometry, dynamic light scattering, and spectroscopy), denaturation of hen egg white lysozyme (HEWL) by treatment with a combination of dithiothreitol (DTT) and guanidine hydrochloride (GdnHCl) has been investigated. The denaturing solutions were selected so that protein denaturation occurred with aggregation (Tris-HCl pH = 8.0, 50 mM, DTT 30 mM) or without aggregation (Tris-HCl pH = 8.0, 50 mM, DTT 30 mM, GdnHCl 6 M) and can be evaluated after 60 min of treatment. It has been found that denatured by solution with 6 M GdnHCl lysozyme completely loses its enzymatic activity after 30 min and the size of the protein molecule increases by 1.5 times, from 3.8 nm to 5.7 nm. Denaturation without of GdnHCl led to aggregation with preserving about 50% of its enzymatic activity. Denaturation of HEWL was examined using interferometry. Previously, it has been shown that protein denaturation that occurs without subsequent aggregation leads to an increase in the refractive index (Δ*n* ~ 4.5 × 10^−5^). This is most likely due to variations in the HEWL–solvent interface area. By applying modern optical techniques conjointly, it has been possible to obtain information on the nature of time-dependent changes that occur inside a protein and its hydration shell as it undergoes denaturation.

## 1. Introduction

It has been nearly 50 years since Anfinsen’s pioneering papers addressing ribonuclease denaturation and renaturation were published [1,2]. The publications showed that proteins are thermodynamically stable and their native structures are determined by the amino acid sequences. One of the vital goals of biophysical chemistry ever since has been finding answers to two fundamental questions: how protein folding goes on, and how the primary amino acid sequence defines the spatial configuration of a folded polypeptide chain [3].

Protein denaturation and renaturation experiments aim to solve one of the major problems of folding—Levinthal’s paradox, which is concerned with how a protein assumes the native (necessary for its biological function) state, selecting it from the great variety of other possible states in a very short time. There are proteins that fold into the native structure in microseconds [4].

The other challenging issue tackled by researchers is what the transition pathway from the denatured (through the molten globule) into the native state is like. In today’s understanding, there are two generally accepted mechanisms of folding: a classic one, through well-defined transition states [2], and a mechanism where multiple transition states are at work at the same time—“the energy landscape theory” [5]. Recently, a quantum folding mechanism has also been proposed [6].

A quantitative analysis of the role of physical bonds inside a protein and in interactions with the solvent is still one of the key approaches to distinguishing forces responsible for a protein’s conformational stability [7]. The stability of the protein native structure is largely defined by the solvent’s parameters (e.g., pH, temperature, the ionic force of the solution, and its chemical composition) [8]. Obviously, changes in these parameters influence the different kinds of bonds that ensure the stability of protein molecules [9]. The principle study method in this area is the evaluation of the conformational change caused by the exposure of the protein globule to different denaturing chemical agents and physical impacts [10]. Hence the reason the majority of studies focus on the processes involved in protein denaturation, effects of different chemical compounds or physical stressors on denaturation, methods of studying the processes involved in denaturation, and so on [11].

Studies of denaturation processes are often of multidisciplinary character [12]. This is because of their relevance in different fields of human activity. For example, some proteins are used in food and pharmaceutical industries as antimicrobial agents. Elucidation of the structure of such proteins when exposed to thermal or chemical stimulation is important—in terms of changes in their antibacterial activity [13]. On the other hand, the loss of the native state does not only lead to inactivation of the protein, but also to protein aggregation, which may cause pathological changes to the organism, ranging from spongiform encephalopathy to systemic amyloidoses [14].

The folding of a polypeptide chain and denaturation are studied in different objects, including albumins [15], globulins [16], pleomorphic proteins [17], peptides [18], and so on. Historically, enzymes have been the most convenient proteins for use in studies of denaturation processes. Enzymes only exhibit their activity when in the native state, so any changes in an enzyme’s structure are easy to control by observing changes in its activity [19].

According to Google Scholar (search query: “denaturation, enzyme”), there are now over 400 thousand papers written in English that address the topic of enzyme denaturation. Of those, 100 thousand are dedicated to lysozyme denaturation (search query: “denaturation, lysozyme”). There have been studies of lysozyme denaturation caused by physical stresses: high [20] or low [21] temperature, pressure [22], pH [23], ionic force [24], and ultrasound [25]. Abundant data have also been collected that address lysozyme denaturation provoked by detergents [26] or stabilizers [27].

Over the past years, the perception of the protein native state as something rigid and distinctly defined has been somewhat “softened”. This is not only associated with increasingly greater attention paid recently to “more flexible”, intrinsically disordered proteins, but also with newly obtained data on how proteins function in the absence of a specific structure [28]. Basically, this shift in the paradigm has been due to the development of new techniques applied in studies of protein structure, those not associated with protein crystallization, which is necessary for X-ray diffraction analysis.

This paper addresses studies of lysozyme denaturation and aggregation caused by the combined action of dithiothreitol and guanidine hydrochloride—those conducted using optical techniques. Interferometry, used as a novel technique to study the hydration shell of proteins [29] together with dynamic light scattering and spectroscopy provide a comprehensive insight into the change dynamics of the protein state in a solution in the course of denaturation or aggregation.

## 2. Results

### 2.1. The Enzymatic Activity of Lysozyme

**Figure 1:**
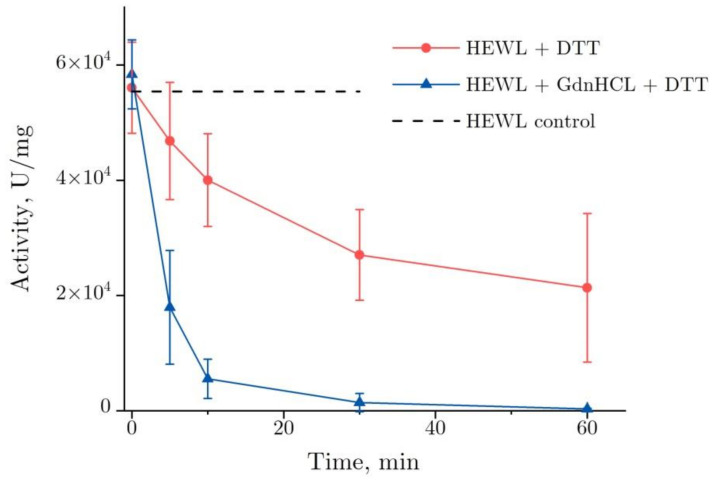
Changes in the enzymatic activity of lysozyme (4 mg/mL) in the course of its denaturation in GdnHCl and DTT (lower curve) and in DTT alone (upper curve) (pH = 8.0, 20 °C). The data are presented as the mean and standard deviation calculated for three test runs. HEWL, hen egg white lysozyme.

Figure 1 shows the dynamics of lysozyme enzymatic activity after the co-action of DDT and GdnHCl. The enzymatic activity of lysozyme was observed to decrease dramatically in the GdnHCl and DTT solution after 10 min—to about 10% from the baseline value, from 5.8 × 10^4^ to 6 × 10^3^ U/mg, falling to zero after the subsequent 50 min. When denatured by DTT, lysozyme demonstrates a gradual reduction of its enzymatic activity—by approximately 60%, from 5.6 × 10^4^ to 2.1 × 10^4^ U/mg, as observed after 60 min.

Two specific features are noteworthy. One of them was an increase in variance observed in enzymatic activity during denaturation by DTT between 30 and 60 min. In three test runs, the enzymatic activity decrease was within 15 and 80%, as observed after 60 min. That was the main source of inaccuracy in the enzymatic activity measurements. The other one was the enzymatic activity of lysozyme at the starting point (0 min, Figure 1). That has to do with the lysozyme enzymatic activity increasing only insignificantly shortly after the addition of denaturing solutions. That was particularly the case for the addition of the GdnHCl and DTT solutions, following which the increase was 5%.

### 2.2. Dynamic Light Scattering Molecules of Lysozyme, Dithiothreitol, and Guanidine Hydrochloride

**Figure 2:**
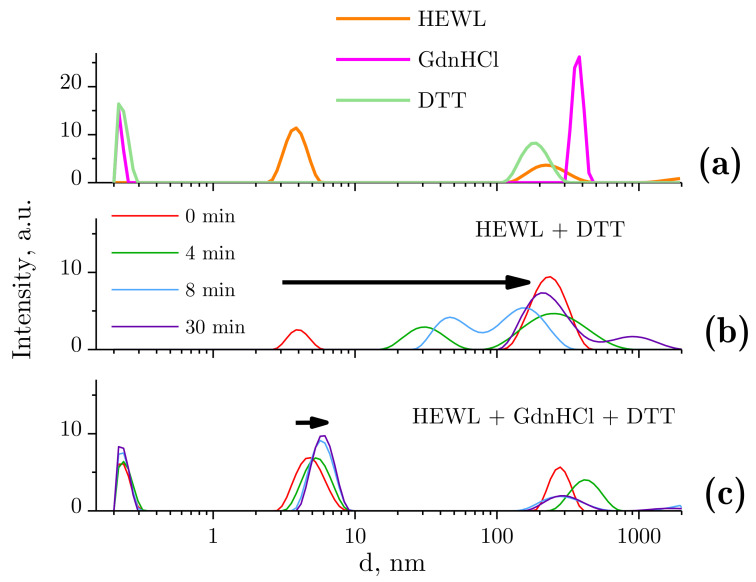
Ordinate axis: peak intensity in arbitrary units, abscissa axis: hydrodynamic diameter d. The data were obtained using dynamic light scattering (DLS). (**a**) Sizes of molecules and molecular aggregates in the following solutions: 4 mg/mL HEWL, 6 M GdnHCl, and 30 mM DTT in 50 mM Tris-HCl (pH = 8.0); (**b**) dynamics of lysozyme molecule sizes with DTT treatment; (**c**) dynamics of lysozyme molecule sizes with GdnHCl and DTT treatment.

Dynamic light scattering was used to evaluate changes in the typical size distribution of lysozyme species following treatment with GdnHCl and DTT solutions (Figure 2). Before the beginning of the test runs, typical size distributions were determined for all tested compounds (Figure 2a). The native lysozyme molecule size was measured to be 3.85 nm. The molecular sizes of GdnHCl and DTT were about 0.2–0.3 nm. The peaks appearing second in the graph (Figure 2a), with the maxima at 200–400 nm, could be attributed to the presence of aggregates, gas nanobubbles, impurities, and so on. Importantly, the quantity of these particles is lower by 18 orders of magnitude, as compared with GdnHCl or DTT, and by 8–10 orders of magnitude, as compared with HEWL. Therefore, the influence of the above-mentioned particles on the protein denaturation and aggregation process can be disregarded. It should be pointed out that the distributions demonstrated for solutions of neat HEWL, GdnHCl, or DTT are stable and remain unchanged after 60 min.

The DLS results are often expressed as particle volume distributions. We present our DLS data as intensity values rather than volume values because the latter option involves the peak related to HEWL (Figure 2c) being invisible owing to the highly concentrated GdnHCl solution. It is important that the denaturing solution (GdnHCl and/or DTT) contains no particles of 2–10 nm in diameter, which makes the DLS technique applicable for tracking denaturation-associated changes in the state of HEWL.

Figure 2b shows the change observed in the size distribution of lysozyme treated with DTT. The hydrodynamic diameter of HEWL molecules was unchanged (3.85 nm) immediately after the addition of DTT. However, a few minutes later, the solution contained no single HEWL molecules, i.e., aggregation was in progress. Subaggregates (dozens of nanometers in size) and aggregates (sized at around 250 nm) were observed. After 30 min, there were no subaggregates left, with only aggregates of the typical size (250 nm) remaining and micron-size aggregates occurring.

Figure 2c shows the change observed in the size distribution of lysozyme treated with the GdnHCl and DTT solution. The hydrodynamic radius of HEWL was increased from 3.8 to 4.7 nm immediately after placing the protein in the denaturing solution (GdnHCl and DTT). After approximately 10 min of continuous increase, it was stabilized, without any meaningful changes observed (5.7 nm).

**Figure 3:**
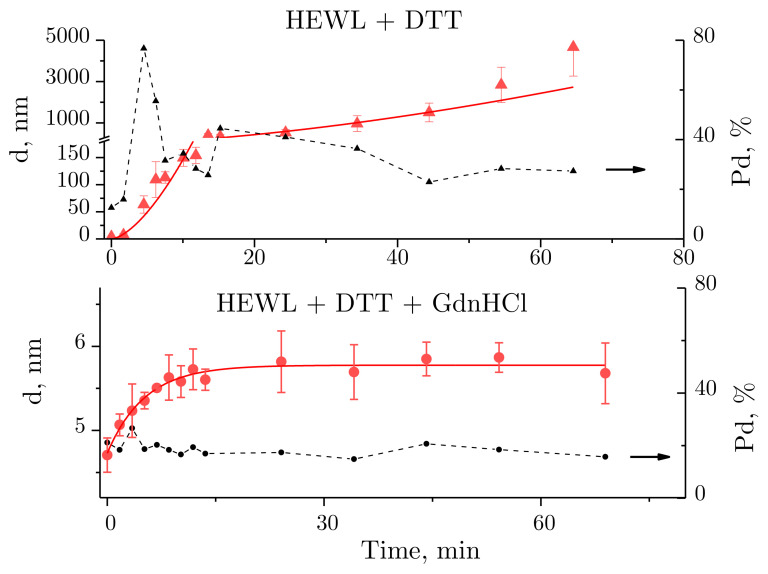
Changes observed over time in the size of molecules and molecular aggregates of lysozyme (red lines) and solution polydispersity (Pd, %) (black dashed line). The data are expressed as means and standard deviations.

For better insight into the hydrodynamic radius changes of lysozyme in the course of its denaturation, curves are shown of hydrodynamic radius and polydispersity values plotted against time of exposure to the denaturing solutions (Figure 3). A power fit in the form of $y=3.9\cdot x^{1.6}$ (for the HEWL + DTT solution) and an exponential function in the form of $y=5.8-e^{\left(-x/5.2\right)}$ (for HEWL + GdnHCl + DTT) are provided. The functions were selected based on their ability to fit the measured data with a minimum standard deviation.

For HEWL denatured by DTT, the mean size of aggregates was observed to increase with time. Noteworthily, the size of aggregates was difficult to determine to sufficient precision at some time-points, as the mean size was calculated from the intensity of multiple closely spaced peaks (e.g., Figure 2b, blue and purple lines). In this case, the size of aggregates was obtained by averaging the sizes of the appearing peaks, each averaged using a weight factor equivalent to the integrated peak intensity. The points in Figure 3 were fitted using a power fit. The size of lysozyme was almost unchanged within the first two minutes, which was followed by aggregation, where particles reached micron sizes after 30 min. No meaningful aggregation was observed for HEWL denatured by the GdnHCl and DTT solution (Figure 2c and Figure 3).

Solution polydispersity (Pd) is an important parameter to consider when calculating particle sizes obtained using DLS. A protein solution is assumed to be monodisperse, i.e., incorporating particles of identical size, at Pd ≤ 20% [30]. Then, the particle sizes can be calculated well enough from the autocorrelation function. For the HEWL + DTT solution (Figure 3 above), Pd < 20% was observed within the first few minutes of the experiment, followed by Pd > 20% for the rest of the experiment. For the HEWL + GdnHCl + DTT solution (Figure 3 below), the following polydispersity values were observed throughout the experiment: Pd ~20% (except for one outlier). Therefore, the size of the protein globule calculated from the autocorrelation function for HEWL denatured by GdnHCl and DTT is correct.

**Figure 4:**
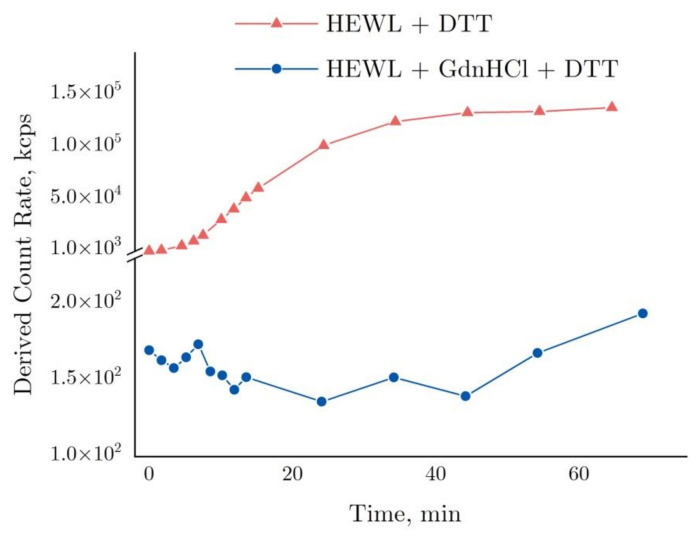
Derived count rate based on DLS data for the denaturation of 4 mg/mL HEWL in 50 mM Tris-HCl (pH = 8.0) with 30 mM DTT or with 6 M GdnHCl and in 30 mM DTT.

The graph in Figure 4 shows time-dependent changes in the derived count rate (DCR). This parameter essentially indicates the number of photons in kilocounts per second (kcps) scattered in a sample and detected by a sensor positioned at 173° towards the laser source axis. To attenuate as appropriate the strong photon flux in Zetasizer Nano ZS, attenuators are utilized. With attenuators applied, the DCR is considered to be equal to its estimated value, i.e., the software uses the attenuation factor and calculates the photon flux in such a way as though no attenuators have been used.

As shown by the graph in Figure 4, the DCR observed for the HEWL + DTT reaction was increased to >10^5^ kcps after 40 min, remaining almost unchanged thereafter. The graph is essentially the same as the dynamics of HEWL molecular aggregation appearing in Figure 3 above, except for a few points around 60 min. In contrast, the DCR remained almost unchanged (120–175 kcps) in the HEWL + DTT + GdnHCl reaction as compared with the aggregation in DTT.

### 2.3. Absorbance Spectra of Lysozyme and Denaturing Solutions

**Figure 5:**
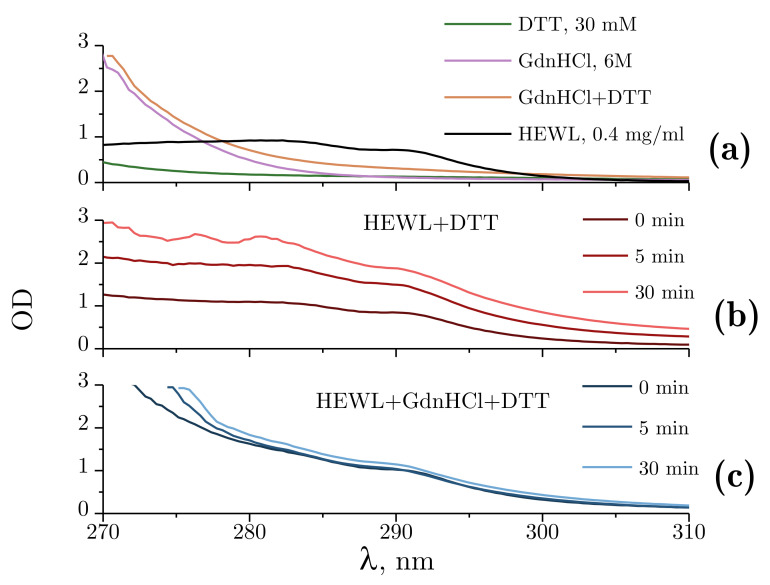
Absorbance spectra within 270–310 nm. (**a**) Absorbance spectra of Tris-HCl (pH = 8.0, 50 mM): DTT (30 mM); GdnHCl (6 M); GdnHCl (6 M) + DTT (30 mM); and 0.4 mg/mL HEWL. (**b**) Optical density changes during the denaturation of lysozyme by DTT at 5 min and 30 min after the mixing of the reagents. (**c**) Optical density (OD) changes during the denaturation of HEWL by GdnHCl and DTT at 5 min and 30 min after the mixing of the reagents. Here, 50 mM Tris-HCl is used as a reference solution.

Figure 5a demonstrates the absorbance spectra obtained for DTT alone and GdnHCl alone, the combination of DTT and GdnHCl, and for 0.4 mg/mL HEWL. The spectra were found to remain unchanged for at least 60 min. Of all the test compounds examined, only the 4 mg/mL protein solution and 6M GdnHCl intensively absorbed ultraviolet radiation at 280 nm, with the absorbance of GdnHCl being nearly half that of lysozyme.

Figure 5b shows the changes in optical density observed during the denaturation of HEWL by DTT. A proportional increase in optical density can be observed throughout the experiment across the spectral range examined. This kind of change points to an increased scattering component, which, within the experiments described, could only be attributed to lysozyme aggregation.

The changes in optical density observed upon denaturation of HEWL under the influence of GdnHCl and DTT are shown in Figure 5c. Changes were only found for the shorter wavelength part of the spectrum, with no significant changes occurring between 300 and 310 nm. The change in the absorbance spectrum observed is associated with internal rearrangements in lysozyme.

**Figure 6:**
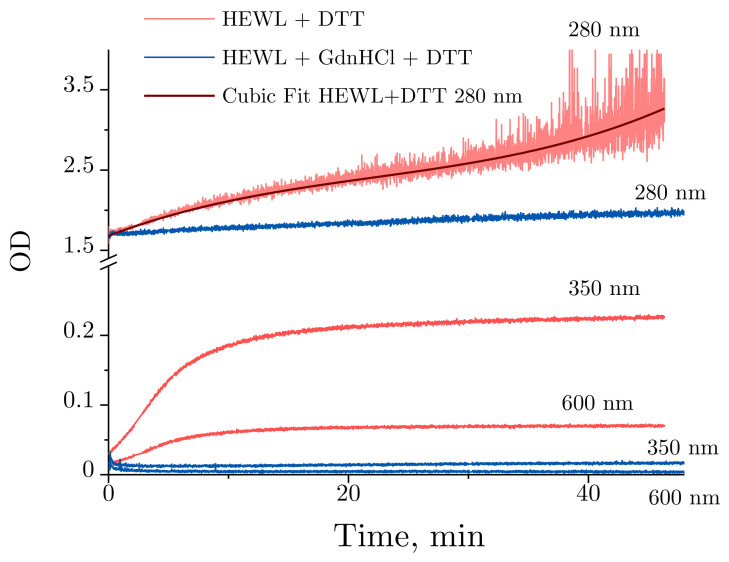
Time-dependent OD (optical density) changes obtained for 0.4 mg/mL HEWL denatured in 50 mM Tris-HCl (pH = 8.0) with 30 mM DTT (red) or 6 M GdnHCl and 30 mM DTT (blue) at 280, 350, and 600 nm. The OD changes of lysozyme treated with DTT at 280 nm against time were fitted with a cubic polynomial.

Figure 6 provides a more detailed illustration of the optical density dynamics observed for HEWL denaturation. Slight changes are observed at 280 nm for HEWL denatured by GdnHCl + DTT throughout the experiment, with no significant changes detected in lysozyme treated with GdnHCl and DTT. Contrarily, the optical density was significantly increased over the first 10 min for HEWL treated with DTT between 350 and 600 nm. After 10 min, the optical density between 350 and 600 nm continues to increase at a significantly slower rate. Normally, increased optical density in the visible region of the spectrum observed for unstained proteins indicates their aggregation. Of note are the time-dependent changes in optical density obtained for HEWL denatured by DTT at 280 nm. The optical density was observed to increase steadily throughout the experiment, with the increase being most pronounced during the first 10 min.

### 2.4. Interferometry of Lysozyme and Denaturing Solutions

**Figure 7:**
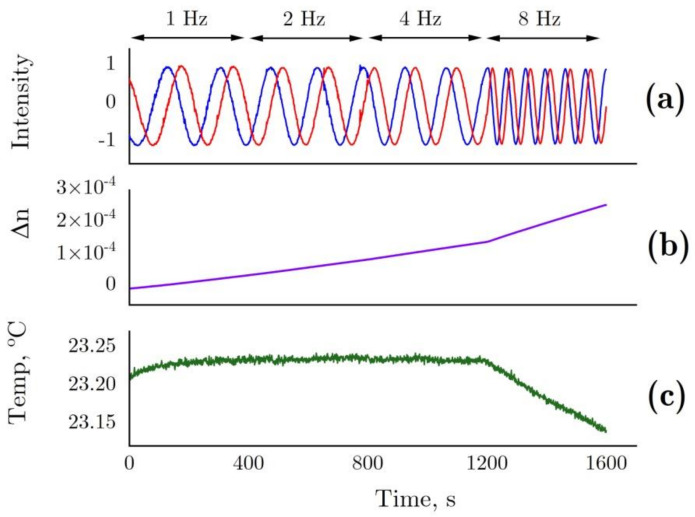
(**a**) The changes in sensor intensity due to pattern shift in the interferometer for 6 M GdnHCl in 50 mM Tris (pH = 8.0) in the test cell and 50 mM Tris (pH = 8.0) in the control cell. (**b**) Refractive index determined from the shift in the interference pattern. This parameter presented with allowance for temperature differences between the cells. (**c**) Temperature in the test cell. In the test cell, the rotation frequency of the non-magnetic mechanical stirrer changed after every 400 s, 1–2–4–8 Hz. In the control cell, the rotation frequency of the stirrer remained unchanged (1 Hz).

Data related to interferometry are presented below. Figure 7 shows the changes in sensor intensity due to pattern shift (Figure 7a) and calculated refractive index changes (Figure 7b) in the optical cell filled with 6 M GdnHCl 50 mM Tris (pH = 8.0) as compared with the control cell containing 50 mM Tris (pH = 8.0). During the measurements, the rotation frequency of the non-magnetic mechanical stirrer was doubled after every 400 s, from 1 Hz to 8 Hz. The stirrer inside the control cell was operated at a constant frequency (1 Hz). The increasing rotation frequency of the stirrer most probably enhanced water evaporation, leading to refractive index changes as a result of a slightly increased GdnHCl concentration in the solution (Figure 7b). This being due to water evaporation is confirmed by a lower temperature observed at 8 Hz (Figure 7c)

**Figure 8:**
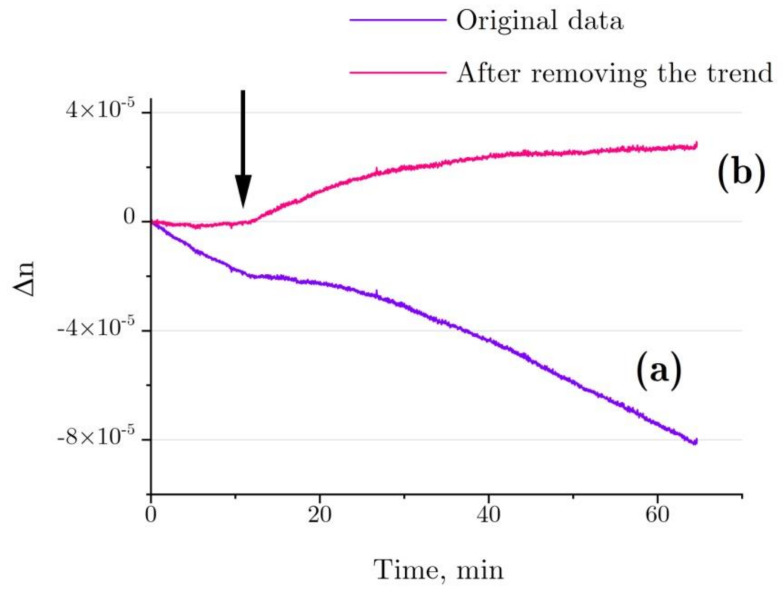
(**a**) Typical refractive index change for the denaturation of 4 mg/mL HEWL in 6 M GdnHCl, 30 mM DTT, and 50 mM Tris in the test cell, as compared with 6M GdnHCl, 30 mM DTT, and 50 mM Tris in the control cell. (**b**) After deduction of the trend associated with water evaporation. The arrow indicates the point of time at which lysozyme was added into the test cell (with Tris added into the control cell simultaneously).

Figure 8 shows the refractive index changes for the denaturation of 4 mg/mL HEWL in 6 M GdnHCl, 30 mM DTT, and 50 mM Tris in the test cell in comparison with 6 M GdnHCl, 30 mM DTT, and 50 mM Tris in the control cell. According to the graph, the refractive index is observed to change even before the addition of HEWL, which is most likely to have been due to slight evaporation differences between the optical cells (Δ*n* ~ 20–30% from the test run using a cell with GdnHCl and another cell containing Tris, Figure 7b, stirrer rotation frequency: 1 Hz). Most probably, the evaporation difference between the optical cells is to do with different geometry of placement of the stirrers inside the cells.

**Figure 9:**
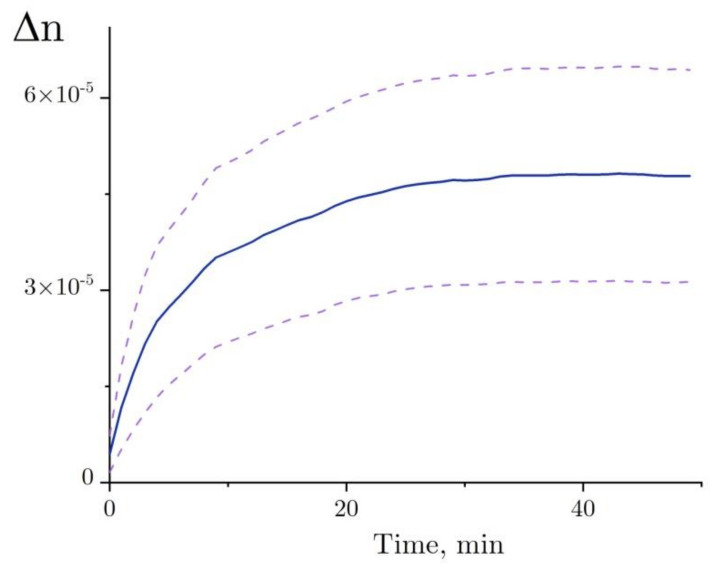
The change in the refractive index of the lysozyme solution (4 mg/mL) during denaturation in 50 mM Tris-HCl buffer (pH = 8.0) with the addition of 6 M GdnHCl and 30 mM DTT at room temperature. The upper and lower dashed lines of the standard error of mean are calculated from six experiments. The figure is plotted using the results published in [31].

A trend in Δ*n*, as observed before the addition of lysozyme, was calculated for each independent test run of the refractive index experiment with the denaturation relation (Figure 8, line a), and then it was deducted from the refractive index profile (Figure 8, line b). The refractive index of lysozyme was increased as observed during the exposure to HEWL + DTT + GdnHCl (Figure 9). This result was first published in the article [31]. Here, we show in detail how it was received (Figure 7 and Figure 8). The refractive index is observed to increase over the first 20–25 min after the start of denaturation—to about 4.5 × 10^−5^, remaining almost unchanged thereafter, which is consistent with the enzymatic activity and DLS observation. It was impossible to study lysozyme denaturation by DTT using interferometry, as the solution quickly became turbid as a result of protein aggregation.

## 3. Discussion

The denaturation of HEWL in two buffers, DTT + Tris and GdnHCl + DTT + Tris, was studied using different optical techniques. It is known that the catalytic activity of lysozyme decreases in the presence of DTT [32] or GdnHCl [33]. DTT and GdnHCl solutions are often used to denature a protein for protein renaturation studies [34]. We have shown that co-treatment with DDT and GdnHCl (Figure 1) reduces the catalytic activity of lysozyme in a shorter time than DDT (Figure 1) or GdnHCl [33], respectively. As shown by Figure 1, the variance in enzymatic activity measurements increases between 30 and 60 min, as observed for the reaction with DTT. After 60 min, the enzymatic activity was observed to decrease within the range of 15 to 80%. We think the increase in variance could have been caused by the formation of aggregates, which have different physicochemical properties. As a result, the activity of proteins can vary significantly in such structures [35]. The protein’s enzymatic activity was increased compared with the control in all test runs, as observed immediately after the addition of DDT and GdnHCl (starting point). The following mean values were obtained: 5.8 × 10^4^ ± 0.6 × 10^4^ U/mg (with the treatment in DDT and GdnHCl) versus 5.5 × 10^4^ ± 1.5 × 10^4^ U/mg. Most probably, that was owing to protein aggregates present in a small quantity in the HEWL solution and that degrade upon exposure to GdnHCl. On the whole, the presence of aggregates has a negative impact on enzymatic activity. These initial aggregates may not be visible in the DLS method. If the aggregates are small, consisting of several lysozyme molecules, and there are few of them, then their peak will not be separated from the peak of monomolecular lysozyme. Another explanation is that the denaturant at the first moment changed the structure of the protein, so that the active center became more accessible to the substrate. Another likely explanation could be the spontaneous lysis of *M. lysodeikticus* induced by the low concentrations of GdnHCl (~2.3 mM) and DTT (~12 μM) left after the dilution of stock solutions used for testing the activity of HEWL (the stock solution was diluted 2600 times).

In addition, DDT is an amphiphilic molecule. These types of molecules self-assemble into nanoassociates in aqueous solution [36]. One molecule of DTT has two SH groaboves, each of which can form an S–S bridge to link to another DTT molecule. Presumably, DTT could be able to form covalently linked chains in solutions. However, if such nanoasspociates actually were to assemble, they would not be larger than 0.2–0.3 nm in size (Figure 2a).

Dithiothreitol, a strong reducing agent, leads to the disrabovetion of S–S bonds in proteins and peptides. HEWL has four disulfide bonds. In the presence of DDT and without GdnHCl, the protein slowly, for over 2 min, loses its native state (d = 3.85 nm) and then aggregates (Figure 2b, Figure 3 above, Figure 4 above). The determined hydrodynamic radius is consistent with the size of the lysozyme molecule of about 3 × 3 × 4.5 nm, obtained from X-ray diffraction analysis [37] or from pulsed field gradient nuclear magnetic resonance, where the hydrodynamic radius was 2.05 nm [38].

In solutions at neutral pH, DTT can be found in either the oxygenated or the deoxygenated form. Improper disulfide bonds in the lysozyme molecule may produce more than 100 isomers [39]. On the other hand, disulfide bonds play a significant, yet understudied role in protein aggregation. It is known that, in 50% of cases, amyloid aggregation is caused by the disrabovetion of disulfide bonds [39,40]. Despite aggregation, the activity of lysozyme, though reduced to nearly 40%, is not lost completely following 60 min of treatment with DTT, pointing to the active site of the enzyme being undamaged following the disrabovetion of S–S bonds.

The protein is not reactive in the presence of GdnHCl in the denaturing solution. Once the denaturing solution is added, HEWL increases in size to d = 4.7 nm, and, after 15–20 min, it reaches the size of 5.7 nm (exponential curve, Figure 4 below). Notably, the time-dependent denaturation observed with exposure to HEWL + GdnHCl + DTT is consistent with the data obtained using DLS (Figure 4) and using interferometry (Figure 9) and is largely similar to the enzymatic activity (Figure 2), though each of the techniques employed describes the reaction in a unique way. Furthermore, aggregation is easy to detect by measuring scattering intensity (the derived count rate, Figure 5), or using absorption spectroscopy at 350 nm and 600 nm (Figure 6). Lysozyme has been shown to increase in size, when in the denatured state, by 146% from its native size. The consistent results obtained correlate with previous data provided by other authors. For instance, in one of the above-mentioned papers [38], the change in the hydrodynamic radius of denatured lysozyme (8 M Urea, pH = 2) was 169%, with 20.5 Å in the native state and 34.6 Å in the denatured state. Minor differences observed in molecular size following denaturation could be due to the varying pH and physicochemical properties of denaturing agents. The denaturation process can also be influenced by the concentration of the denaturing agent. In particular, in the paper by [41], even the use of the denaturant concentration of 8 M instead of 6 M led to changes in the size of denatured bovine serum albumin.

Significant changes were observed for HEWL treated with DTT across the wavelengths examined. However, pronounced increases in optical density were only recorded for the first 10 min between 350 and 600 nm (Figure 5 and Figure 6). Increased optical density in the visible region of the spectrum observed for unstained proteins indicates their aggregation [42]. Peculiar optical density dynamics was obtained for HEWL denatured by DTT at 280 nm. The optical density increased steadily throughout the experiment. We attribute this to an increased concentration of the oxidized form of DTT in the solution, which has its absorbance peak at 283 nm [43]. The increases in optical density were accompanied by increased variance in the measured variables. That is a characteristic feature of the method. At OD = 3, the sample absorbs 99.9% of visible light, with the contribution from the noises of the photoelectric transducer becoming dominant.

Slight optical density changes were detected for HEWL denatured by GdnHCl + DTT at 280 nm throughout the experiment (Figure 6). The minor increase in optical density was probably associated with water evaporation from the optical cell rather than HEWL denaturation, which enhanced the concentration of GdnHCl in the concentrated 6 M solution, through this increasing the OD. Similar effects were noted with interferometry observations. Figure 7b shows increases in the refractive index of 6 M GdnHCl present in the test cell in comparison with water in the control cell. The increase is most likely to have been caused by evaporation. That is confirmed by a higher rate of change observed in the refractive index with an increased rotation frequency of the stirrer, and by a lower temperature recorded in the optical cell with the maximum rotation frequency of the stirrer (8 Hz, Figure 7c). Similar refractive index changes were recorded in the study by [44], where a negligible change in the tested GdnHCl concentration from 6 M to 6.01 led to a refractive index change of 1 × 10^−5^. When the same process was examined at 350 nm and 600 nm, no trends of the kind were detected, as GdnHCl has no absorbance at these wavelengths.

The refractive index of the protein solution was shown to increase with protein denaturation (Figure 8 and Figure 9) [31]. The effect observed during denaturation as a refractive index increase of ∆*n* ~ 4.5 × 10^−5^ was several times the effect observed with proteolysis [29]. This seems odd, as the change observed in the size of the HEWL molecule (from 3.85 nm to 5.7 nm) was to be accompanied by an increase of σ ~2.2 (the molecule simplified as spherical shape), which should lead to a refractive index increase of comparable magnitude. The following explanation could be suggested. In order to simplify the model, the *u* parameter was not changed in the description of the proteolysis process (see Equation (1)). In the case of denaturation, the *δ_c_* parameter (reduction of the density of water present in the protein cavities as compared with bulk water) can vary within a broad range and can correspond to *δ_c_* >> 1% in hydrophobic cavities of about 1 nm in size. This assumption is backed by the results of the study by [45], which used computer modeling to show that the space of about 1 nm between two nano-sized hydrophobic surfaces placed in an aqueous salt solution may contain no water molecules.

At the same time, the reaction occurs in a concentrated (6 M) GdnHCl solution, with four water molecules per molecule of GdnHCl, which may substantially influence the structure of water at the water–protein interface. The publication by [46] describes the changes in the refractive index increment with different buffer solutions selected. The refractive index proved to vary within a broad range—0.153 cm^3^/g for H_2_O to 0.272 cm^3^/g for NaSCN 10 mM HEPES 10 mM—and it was close to the estimated value for the majority of proteins—0.18–0.19 cm^3^/g—in most of the other buffers used [47].

The refractive index measured for lysozyme during its denaturation varied to a great extent in different experiments (Figure 9). The great variance could be due to the different shapes and sizes of denatured lysozyme obtained in different experiments. Thus, the results of DLS demonstrated a difference of 10% in the size of the denature lysozyme between two experiments. Together with this, the great uncertainties in refractive index (lower and aboveper curves, Figure 1) could have been caused by measurement errors associated with the high concentration of GdnHCl in the solution. There was evaporation of water from both the test cell (protein in the denaturing solution) and the control cell (denaturing solution) during the experiment (see above). So, even minor differences in evaporation rate, i.e., an insignificant increase in GdnHCl concentration in one cell compared with the other one, could lead to trends in the refractive index measurements (Figure 8a), making gathering data on the protein states difficult.

In fact, both the example with proteolysis from the study by [29] and our protein denaturation experiments have shown that increases observed in the refractive index during the reaction depend on the change in the area of the protein/peptide/solvent interface. The effects of the interface area between the protein and the solvent on the refractive index of the protein solution were also noted in the study by [48], which demonstrated differences between the estimated refractive index of a protein and its measured value by testing a number of proteins. The identified differences were greater with larger areas of the interface with the solvent. The above effects suggest that the refractive index can be used to study the structure of proteins in solutions.

Recently, there has been growing interest in using interferometry to study the properties of biomolecules. The Mach–Zehnder scheme is especially widely used, and it is used not only in single-beam installations, but also for studies in a wide spectral range [49]. The data obtained using interferometry are in good agreement with other optical methods, such as circular dichroism [50], fluorescence [51], infrared spectroscopy [52], and so on. This suggests a great potential for using interferometry as a method that can provide new information about biomolecules.

## 4. Materials and Methods

### 4.1. Materials

Lysozyme was derived from hem egg-white (>20,000 U/mg, Am-O633, Amresco, Solon, OH, USA), *Micrococcus lysodeikticus* (lyophilized cells, ATCC No. 4698, Sigma-Aldrich, St. Louis, MI, USA), DL-Dithiothreitol (DTT, D3483123, Dia-M, Moscow, Russian Federation), and GdnHCl (100005172, Dia-M, Moscow, Russian Federation), Tris (Panreac, Barcelona, Spain). The water used for the experiments was produced by distillation and deionization to a resistance of >10 MΩ/s.

### 4.2. Denaturation of the Protein

Lysozyme chemical denaturation was performed using two solutions. One solution contained 50 mM Tris-HCl, pH 8.0, as implemented above with 6 M GdnHCl and 30 mM DTT. The other solution was 50 mM Tris-HCl with 30 mM DTT. The protein concentration of 4 mg/mL was used for all the methods, except UV/visible absorbance spectroscopy; the concentration was reduced to 0.4 mg/mL because of a high absorbance of lysozyme. The denaturation experiments were performed at 22–24 °C and used the following solution volumes: 15 mL for interferometry, 3 mL for UV/visible absorbance spectroscopy, 1.25 mL for DLS, and 0.5 mL for the enzyme assay.

### 4.3. Enzyme Assays of Lysozyme

The activity of lysozyme was examined using the lysis of *M. lysodeikticus* cells, at room temperature, as described in [53]. Here, 10 μL of lysozyme was collected from the denaturing solution, diluted 100 times in 50 mM Tris-HCl, and added, at 100 μL and a concentration of 40 μg/mL, to 2.5 mL of the micrococcus diluted in 20 mM of K_2_HPO_4_ (pH = 7.0) to an OD of about 0.7–0.8 (λ = 450 nm). The activity was measured by decreases in OD at the same wavelength with a spectrophotometer (PE-5300VI, Ekros, Russia) for the first two minutes after the addition of lysozyme. The activity of lysozyme following denaturation was compared to that of native lysozyme, which had undergone identical dilution procedures, with Tris used instead of GdnHCl and DTT. Measurements of lysozyme activity observed during denaturation were performed as three experiments on different days.

### 4.4. UV/Visible Absorbance Spectroscopy

An ISS-UV/VIS Spectrometer (Ocean Optics Inc., Orlando, FL, USA) was used for the UV/visible absorbance spectroscopy experiments. To examine the time dependence of OD changes, a non-magnetic, mechanical stirrer was fitted in a standard optical cell, which allowed performing lengthy measurements without additional stirring, normally used to prevent precipitation of heavy molecules and/or aggregates.

### 4.5. Dynamic Light Scattering

Zetasizer Nano ZS (Malvern Panalytical Ltd., Malvern, UK) was used to measure dynamic light scattering. The measurements were performed at 25.0 °C in a polystyrene optical cell. The particle size was calculated using Zetasizer Software 7.13 (Malvern Panalytical Ltd., Malvern, UK). To calculate the size of protein globules studied by DLS, the viscosity value and the refractive index of the solution are necessary. While the viscosity and refractive index calculated for 30 mM DTT and 50 mM Tris are close to the values of water, these characteristics of 6 M GdnHCl differ meaningfully. The viscosity η = 1.613 and refractive index were obtained for 6 M GdnHCl from [54] and from [44], respectively. The contribution from 30 mM DTT and 50 mM Tris was neglected as it was lower than 1% as compared with the contribution from 6 M GdnHCl in the denaturing solution GdnHCl and DTT.

### 4.6. Laser Interferometer

A high-precision Mach–Zehnder interferometer using a red laser diode (GH0631IA2GC 638 nm CW 185 mW, Sharp, Tokyo, Japan) was used for the experiments [29]. A beam power of about 1 mW was applied. For each experiment, the control cell and the test cell, where the reaction was generated, were fitted with platinum temperature sensors (HEL-705, Honeywell, Charlotte, NC, USA) to record the temperature, as well as non-magnetic mechanical stirrers. The stirrers were operated at a frequency of about 1 Hz. It was important to use the stirrers during the interferometry process; otherwise, the interferometric pattern was distorted by the inhomogeneities in the highly concentrated GdnHCl solution, interfering with the refractive index measurements. At the same time, the low stirring speed did not influence the temperature in the cell or the denaturation rate.

There was no difference between the denaturation speed measured by the enzymatic activity in a stationary 0.5 mL Eppendorf tube and the values in the interferometer cells (filling volume: 15 mL), also measured by the enzymatic activity. Six test runs were performed in the interferometer on different days at an average ambient temperature of 22.8 ± 0.8 °C.

### 4.7. Evalution of Hydration Shell Parameters Based on Refractive Index Variations in the Solution

Current theoretical refractive index evaluations in protein solutions are based on the refractive index measured in solutions of the amino acids incorporated into the protein in question [47,55,56]. As the concentration of the amino acids is unchanged in a solution undergoing denaturation, the refractive index change is largely unexpected. However, our previous studies have demonstrated increasing refractive indices in the protein solutions (HEWL and BSA) undergoing proteolysis by pepsin [29,57]. The authors [29] introduced a model and an equation to explain refractive index variations observed during the proteolysis process:

(1) $$\Delta n\left(t\right)=\left(\frac{3\left(R\left(t\right)-R_{0}\right)}{2n_{0}{\left(1-R_{0}\right)}^{2}}\right),\;\;R\left(t\right)=\frac{4\pi }{3}\frac{N\alpha^{\prime }}{W-uqs\sigma \left(t\right)}$$

where $n_{0},\;R_{0}—$ refractive index and refractive constant before the initiation of the reaction; W— “virtual” water volume (assuming all the water had the density of bulk water); $N,\;\alpha^{\prime }$ —quantity and polarizability of water molecules; q,s —quantity and surface area of the protein molecules; $\sigma \left(t\right)$ —change in the total surface area of the peptide as compared with the original protein; and the hydration shell parameter $u=\delta_{h}d+\delta_{c}zr$, where $d,\;\delta_{h}$ —hydration shell width and dimensionless increase in water density in the hydration shell as compared with bulk water, $r,\;\delta_{c}$ —radius of the protein cavity and dimensionless decrease in the density of water in the protein cavities as compared with bulk water, and *z*—a unity-order constant associated with the cavity geometry.

In the paper by [29], the refractive index was increased by ∆*n* ~ 2 × 10^−6^ after 60 min of the proteolysis reaction, and by ∆n ~ 6 × 10^−6^ (estimated value) by the end of the reaction. The increase σ in the total peptide area for HEWL was estimated to be ~2, with u = 2.7 × 10^−3^ nm, which, given the absence of cavities in the peptide and a hydration shell width *d* of one water molecular layer, is equivalent to a water density increase in the protein hydration shell of $\delta_{h}\approx 1\%$ versus bulk water.

## 5. Conclusions

Denaturation and aggregation of hen egg-white lysozyme co-treated with dithiothreitol and guanidine hydrochloride were studied using interferometry, dynamic light scattering, and spectroscopy. When denatured by GdnHCl + DTT, lysozyme loses its enzymatic activity after 30 min, while its hydrodynamic radius increases from 3.8 nm to 5.7 nm. Where denaturation is induced by DTT alone, the protein retains approximately 40% of its enzymatic activity, as observed after 60 min of exposure. Protein aggregation is observed shortly after the start of the reaction (within 10 min), and then subaggregates of dozens of nanometers in size assemble, aggregate into larger particles (200–400 nm) after 30 min, and reach micron sizes after 60 min.

UV/visible absorbance spectroscopy has been shown to be capable of tracing all the above processes. Although, in the case of solutions with 6 M GdnHCl, spectroscopic measurements are complicated owing to the high absorption in the UV region (λ < 280 нм).

Denaturation of HEWL was examined using interferometry. Previously, it has been shown that protein denaturation that occurs without subsequent aggregation leads to an increase in the refractive index (Δ*n* ~ 4.5 × 10^−5^, Figure 9) [31]. Here, we show in detail how it was received (Figure 7 and Figure 8). The proposed model explains how the increase in the refractive index depends on changes in the HEWL–solvent interface area.

Connections were established between measured parameters of protein solutions examined by DLS, spectroscopy, and interferometry. Interferometry gives information not about the structure of a protein molecule, but rather about the hydration shell around it. In this regard, interferometry does not compete with other generally accepted research methods such as 2D-IR, spectrofluorometer, circular dichroism, and so on, but rather provides new information about the nature of the interaction of a protein with a solvent. The methods appear to be complimentary and provide information on the nature of changes that occur not only in the structure of a protein, but also in its hydration shell.

## Data Availability

Not applicable.
